# Human papillomavirus (HPV) prevalence and types among Turkish women at a gynecology outpatient unit

**DOI:** 10.1186/1471-2334-9-191

**Published:** 2009-11-30

**Authors:** Polat Dursun, Süheyla S Senger, Hande Arslan, Esra Kuşçu, Ali Ayhan

**Affiliations:** 1 Baskent University School of Medicine, Departments of Obstetrics & Gynecology, Ankara, Turkey; 2 Departments of Infectious Disease, Ankara, Turkey

## Abstract

**Background:**

Human Papillomavirus (HPV) is a well-known pathogen for lower genital tract neoplasias, yet little is known regarding HPV prevalence in Turkey. The aim of this study was to investigate the prevalence of HPV DNA and to determine HPV types distribution among women with normal and abnormal cytology.

**Methods:**

A total of five hundred seven (n = 507) women were retrospectively evaluated between 2004-2008. Conventional polymerase chain reaction was used to detect the presence of HPV types in cervicovaginal samples obtained from patients during gynecologic examination.

**Results:**

One hundred four (n = 104) of the women were excluded from the study because of the incomplete data and a total of 403 women were used for the final analysis. There were, 93 (23%) women with cytologic abnormality and 310 (77%) women with normal cytology. Overall, 23% of the women was HPV positive. The overall prevalence of HPV in women with abnormal Pap smears was 36% (93/403), of which in ASCUS 22%, LSIL 51% and HSIL 60%. Also, HPV DNA was positive in 20% of the women with normal cervical cytology. The most common HPV types in cytologically normal women were as follows; HPV 16 (36%), HPV 6 (22%) and HPV 18 (13%). The rate of other HPV types were as follows; HPV11 4.4%, HPV45 4.4%, HPV90 4.4%, HPV35 2.2%, HPV67 2.2%, HPV81 2.2%, and multiple type HPVs 8.9%. The most common HPV types in cytologically abnormal women were HPV 16 (35%), HPV6 (19%) and HPV18 (8%). The rate of multiple HPV infections in women with normal Pap test was 2.2%.

**Conclusion:**

HPV prevalence and type distribution in this study were similar to that reported worldwide at least in our study population. Hovewer, HPV prevalence was more common compared with previous studies reported from Turkey. This might be related with methodology and hospital based patient accrual and high rate of women with abnormal cytology. Further population based prospective studies are needed to eliminate the drawbacks of our study and to determine nonhospital based HPV prevalence in Turkish women.

## Background

Cervical cancer is the one of the leading cause of cancer-related deaths among women worldwide. It is e estimated 500,000 cases occurring annually and a 50% case fatality rate [1].

According to statistics of Ministry of Health and GLOBOCAN 2002, cervical cancer is the 9th most common cancer among women in Turkey and ranks 13th among cancer-related deaths, with an estimated 1364 cases occurring annually and more than 50% of fatality rate [2].

Today, it is widely accepted that Human papillomavirus virus (HPV) is the main causal factor of cervical carcinoma. More than 100 HPV genotypes have been described and nearly 20 of them have been associated with cervical carcinoma. It has been reported that HPV prevalence range between 1.4% to 25.6% in the world [3]. Previous studies reported that HPV prevalence between 2% to 6% among the low risk women in Turkey [4-8]. However, these studies generally included small number of cases or performed by using Hybrid Capture I or II. The aim of this study is to investigate the HPV prevalence by using PCR method and to report the rate of HPV infection in women with normal and abnormal cervical cytology.

## Methods

Between January 2004 and August 2008, 503 consecutive sample of women attending gynecological outpatient clinics for regular gynecologic control in Baskent university School of Medicine, Departments of Obstetrics and Gynecology were included to this retrospective analysis. Women were eligible if they had no previous diagnosis or treatment for cervical, vulvar or vaginal cancer and ever sexually active. Also, patients with previous history of chemoradiotherapy for cervical carcinoma were excluded from the analysis. Information on sociodemographic characteristics, medical history and sexual and reproductive behaviour was obtained at the time of the gynecological visit which was filled during the gynecological visit. Samples of exfoliated cervical cells were obtained using a cytobrush. After the preparation of a standard cervical smear, the remaining cells were placed in tubes with 0.9% saline and stored at - 201C until shipment to the our microbiology laboratory. Pap smears were classified according to the Bethesda system by pathology department. HPV DNA detection and genotyping performed by PCR in our microbiology labaratory as described previously [9]. The data were analysed by of the t test, continuity-corrected chi-square method or Fisher's exact test, One way Anova, calculation of prevalence ratio and descriptive statistics as required, and the probability type I error was set at 0.05 (two-tailed). Prevalence ratio for each decads (2th, 4th and 5th) calculated by comparing with 3rd decad using poisson regression with robust variance estimates as described previously [10,11] Statistical analysis performed by SPSS v.11.5.

### HPV DNA detection and genotyping

HPV DNA detection in cervical swabs was conducted by using real-time polymerase chain reaction (PCR) with a commercial kit (Fluorion, Iontek, Turkey). For DNA extraction the QIAamp DNA Mini Kit (Qiagen, Hilden, Germany) was used in accordance with the instructions of the manufacturer. A 150 bp fragment of the L1 gene was amplified using GP5 and GP6 primers. An amplified gene product was identified via melting curve analysis and visualized by incorporation of Sybr Green dye during amplification. HPV genotyping was performed with DYEnamic ET Terminator Cycle Sequencing Kit (Amersham Biosciences Corp., NJ, USA) and ABI PRISM 310 Genetic Analyzer at Iontek Ltd, Turkey.

The study protocol was approved by at Ethical Board of Baskent University, School of Medicine.

## Results

During the four year period, 503 women who attend for regular gynecological examination were analyzed for HPV in our departments. One hundred four of these women were excluded from the analysis because of the incomplete data and a total of 403 women were used for the final analysis.

Mean age of the patients was 37.5 ± 9.3 (range 19-67 y). Ninety percent of the population was premenaposal while 10% was menopausal. There were significant differences with respect to HPV positivity between women under 30 years old and women older than 30 years old (34% vs.20%, p = 0.005) but, there was no significant differences with respect to decads of life and HPV positivity. Also, menopausal status has no effect on HPV positivity. HPV was positive in 24% of the premenopausal women and 17% of the menopausal women (p = 0.323).

There were, 93 (23%) women with cytologic abnormality and 310 (77%) women with normal cytology. Cervical cytologic abnormalities were diagnosed as follows; 42% atypical squamous cell of undetermined significance (ASCUS), 46% low-grade squamous intraepithelial lesion (LSIL), and 11% high-grade SIL (HSIL). Overall, 23% of the women was HPV positive. On the other hand, HPV was positive in the 36% of the women with cytologic abnormalities and this figure was 20% in women without cytologic abnomality. HPV was positive in 22%, 51%, 60% of the ASCUS, LSIL and HSIL respectively (Table 1). The most common HPV types in cytologically normal women were as follows; HPV 16 (36%), HPV 6 (22%) and HPV 18 (13%). The rate of other HPV types were as follows; HPV 11 4.4%, HPV 90 4.4%, HPV 45 4.4%, HPV 67 2.2%, HPV 81 2.2%, HPV 35 2.2%, and mixed type HPVs 8.9%. The most common HPV types in cytologically abnormal women were HPV 16 (35%), HPV 6 (19%) and HPV 18 (8.8%) (Table 2). The prevalence ratio for 2nd, 4th, 5th and 6th decads of life comparing the 3rd decads of life were 1.78, 1.12, 1.14 and 2.07, respectively (Table 3).

**Table 1:** Distribution of cervical cytology and HPV in the study population

Cytology	Mean Age	No. of Specimens N,(%)	Overall HPV (+) %
**Normal**	38.1 ± 9.4 (20-67)	310 (77%)	20%
**Abnormal Cytology**	35.6 ± 8.7 (19-58)	93 (23%)	36%
**ASCUS**	37.0 ± 8.8	39 (42%)	22%
**LSIL**	33.6 ± 8.9	43 (46%)	51%
**HSIL**	38.0 ± 6.9	10 (11%)	60%

**Table 2:** HPV type distribution in cases with normal and abnormal cytology

	HPV Types in All Positive Patients (n = 93)	Normal Cytology (n = 310)	Abnormal Cytology * (n = 93)
**HPV 16**	34%	37%	30%
**HPV 6**	17%	22%	13%
**HPV 18**	9%	14%	5%
**HPV 35**	8%	2%	17%
**HPV 16+18** **(Double HPV)**	8%	7%	5%
**HPV 11**	4%	4%	5%
**HPV 45**	4%	4%	5%
**HPV 31,33,45,52,58** **(Multiple HPV)**	4%	2%	8%
**HPV 31**	4%	-	8%
**HPV 90**	3%	4%	-
**HPV 67**	3%	2%	-
**HPV 81**	2%	2%	4%

* HPV type distribution in all HSILs: HPV 16 in 50%, HPV18 in 25% and HPV 6 in 25%

**Table 3:** HPV prevalence by age in women with normal cervical cytology (n = 310)

Age	HPV (+)/total no. of patients	HPV%	P	PR*
20-29	17/59	28.8	0.29	1.78
30-39	20/124	16.1		
40-49	15/83	18.1		1.12
50-59	7/38	18.4		1.14
>60	2/6	33.3		2.07

* PR: Prevalance ratio for each other age group compared to age 30-39

## Discussion

Today, it is well known that some kind of cancers are closely related with persistent infection of oncogenic types HPV. On the basis of epidemiological and virological studies, HPV is estimated to cause 100% of cases of cervical cancer, 90% of anal cancer, 40% of cancers of the external genitalia (vulva, vagina and penis), at least 12% of oropharyngeal cancers and 3% of oral cancers [12]. It has been estimated that HPV is responsible for 5.2% of all cancers worldwide [13]. Furthermore, Human papillomavirus (HPV) has been established as a necessary cause of cervical cancer and its precursors, including cervical intraepithelial neoplasias grades 2 and 3 (CIN 2-3). Therefore, detection of high-risk HPV is becoming increasingly attractive as a primary screening tool. Also, data for HPV-type distribution is sine qua non to development of new HPV-screening tests and to assessment of the effect of future vaccination on HPV infections of differing severity, but published data about the HPV prevalence in Turkey limited [3-8].

Although Turkey has historically had low incidence rates of invasive cervical cancer, cervical cancer ranks among the 9th most common female malignancies with an age-standardized incidence rate of 4.5 per 100.000. Our country has a more than 70 million population, but, unfortunately, there is no population based studies which investigating HPV prevalence in Turkish women. Previous studies reported that HPV prevalence between 2%-6% among the low risk women in Turkey (Table 4). In contrast, HPV prevalence and type distribution in our study was similar to that reported worldwide. Our HPV prevalence is the highest rate reported from our country so far and probably just reflect the HPV profile of our patients population. We believe this high prevalence may arise from the high rate of women with abnormal cervical cytology, the higher educational status of our patient population [14,15]. hospital based patient accrual and usage of more sensitive HPV evaluation method. As it can be seen in Table 4, prevalence of HPV in Turkey was found 2 times higher in PCR studies compared with hybrid capture studies. Interestingly, Bao et al. reported that HPV prevalence increased with the dates of study publication due to the technological improvement of the HPV measuring methods. This situation may also be generalized to our study as well [16].

**Table 4:** HPV studies reported from Turkey

	N	Method	%
**Ege SSK Hospital, Ýzmir **[4]	1353	HC-II	2.1
**Hacettepe University Hospital, Ankara **[5]	1032	HC-II	4
**Erciyes University Hospital, Kayseri **[6]	230	HC-I	6

**Total**	2615	Mean of HCs	4%

**Numune Hospital, Ankara **[7]	134	PCR	2.2
**Hacettepe University Hospital-2, Ankara **[8]	60	PCR	3.3
**Baskent University Hospital, Ankara (Current study)**	403	PCR	20

**Total**	597	Mean of PCRs	8.5%

HC: Hybride Capture, PCR:Polymerase chain reaction

In 2007, Sanjosé et al. reported that overall HPV prevalence in 157.879 women with normal cervical cytology was estimated to be 10.4% in a meta-analysis of 78 studies [17]. In 2008, Bao et al. reported that overall HPV prevalence was 14.4% in Asia in a meta-analysis of 79 studies which including 25,368 women [16]. In this study, for women with normal cytology/histology, HPV prevalence had a smaller range of 14.0% in southeastern Asia to 14.4% in south central Asia. On the other hand, in an another meta-analysis including the results of 15.613 women, overall HPV prevalence was 8.7%,14.3% and 5.2% for Asia, South America and Europe, respectively [3]. In our study, the rate of HPV positivity in women with normal cervical cytology was 20%. Variations between studies most likely reflect differences in the population studied with respect to risk factors for exposure to HPV and methods of evaluations.

In women with abnormal cytology results, the presence of HPV infection has been reported in 28.8%-61.3% of cases [18-22]. In our study, HPV was positive in 36% of women with abnormal cytology. This low rate of HPV in women with abnormal cytology could be explained by the false evaluation of the Pap test of women which was referred to our instution. HPV infection by multiple genotypes has been reported to occur in 10% to 20% of HPV-positive cases and to show dependence on HPV type, age of the patient, and the presence of cytologic abnormalities [23]. Interestingly, we also found that 8.9% of HPV-infected women has more than one HPV genotype in women with normal cervical cytology while this percentage was 11.8 in women with abnormal cervical cytology (p > 0.05). In other studies, this figures varies between 7% and 23% depending on the cytological diagnosis. Double infection with HPV 16 and 18 was found 6.6% in women with normal cervical cytology. Arora et al. reported the 6.7% of the women with normal cytology had double infection with HPV 16 and 18 [24]. Therefore, Arora et al suggested that the cases who are HPV positive for any of the high risk types but have concurrently negative Pap smear or no visible lesions should be followed up more frequently than the HPV negative cases as they have a substantially greater risk of developing an abnormal smear [24,25]

Some epidemiologic studies reported that a significant reduction in HPV prevalence throughout the third decade of life [26]. It has also been reported that HPV prevalence is between 32-64% in women under 25 years-old women while this figure between 2-4% in women older than 45 years old [27]. Similarly, in our study the rate of HPV infection was 34% in women under 30 years old and this rate was significantly higher than women older than 30 years old. Hovewer, there was no significant differences in HPV prevalence by decads of life in patients with normal cervical cytology (Table 3). This situation also needs to be explanation. On the other hand, it is weel known, HPV-16 is the most prevalent type worldwide. However, the second most prevalent one is HPV-18 in Western countries, whereas it is HPV-58 in Asia [6]. HPV 16, 6 and 18 were the most common three type in our study population as in western countries [23]. On the other hand, HPV prevalence was reported to be 81.0% in women with HSIL. The ten most common HPV types were 16, 58, 52, 18, 33, 51, 31, 56, 35, and 45 in HSIL [16]. Onan et al from Turkey reported that 45% of the women with cervical intraepithelial neoplasia (CIN) had high risk HPV [28]. Inal et al. performed HC-II study in 1353 women from Izmir region which has the highest cervical cancer incidence in Turkey, and reported 2.1% women had positive for HPV. Hovewer, all the women with cytological abnormalities had positive for HPV DNA. Therefore, the authors concluded that "In spite of low general frequency of cervical HPV colonization, there was a strong correlation between HPV and CIN" [4]. In our study, HPV was positive in 36% of the cases with cytological abnormalities.

## Conlusion

In conclusion, although geographicly Turkey is located between Europe and Asia, HPV prevalence and genotypes are similar to those reported from western countries in Turkish women with normal and abnormal cytology. Hovewer, HPV prevalence was more common compared with previous studies reported from Turkey. This might be related with methodology and hospital based patient accrual and high rate of women with abnormal cytology. Further population based prospective studies are needed to eliminate the drawbacks of our study and to determine nonhospital based HPV prevalence in Turkish women.

## Competing interests

The authors declare that they have no competing interests.

## Authors' contributions

PD, conceived of the study, performed analysis and interpretation of data, drafted the manuscript and revised the manuscript. SSS performed the data analysis, revised the manuscript. HA, performed the data analysis, revised the manuscript. EK supervised the study, revised the manuscript. AA, supervised the study and revised the manuscript. All authors read and approved the final manuscript.

## Pre-publication history

The pre-publication history for this paper can be accessed here:

http://www.biomedcentral.com/1471-2334/9/191/prepub
